# Porcine Fetal-Derived Fibroblasts Alter Gene Expression and Mitochondria to Compensate for Hypoxic Stress During Culture

**DOI:** 10.1089/cell.2018.0008

**Published:** 2018-08-01

**Authors:** Bethany R. Mordhorst, Stephanie L. Murphy, Martin Schauflinger, Shirley Rojas Salazar, Tieming Ji, Susanta K. Behura, Kevin D. Wells, Jonathan A. Green, Randall S. Prather

**Affiliations:** ^1^ Department of Animal Sciences, University of Missouri, Columbia, Missouri.; ^2^ Electron Microscopy Core Facility, University of Missouri, Columbia, Missouri.; ^3^ Department of Statistics, University of Missouri, Columbia, Missouri.

**Keywords:** hypoxia, gene expression, mitochondria, fetal fibroblasts, Warburg effect

## Abstract

The Warburg effect is characterized by decreased mitochondrial oxidative phosphorylation and increased glycolytic flux in adequate oxygen. The preimplantation embryo has been described to have characteristics of the Warburg effect, including similar changes in gene expression and mitochondria, which are more rudimentary in appearance. We hypothesized hypoxia would facilitate anaerobic glycolysis in fibroblasts thereby promoting gene expression and media metabolite production reflecting the Warburg effect hallmarks in early embryos. Additionally, we speculated that hypoxia would induce a rudimentary small mitochondrial phenotype observed in several cell types evidenced to demonstrate the Warburg effect. While many have examined the role hypoxia plays in pathological conditions, few studies have investigated changes in primary cells which could be used in somatic cell nuclear transfer. We found that cells grown in 1.25% O_2_ had normal cell viability and more, but smaller mitochondria. Several hypoxia-inducible genes were identified, including seven genes for glycolytic enzymes. In conditioned media from hypoxic cells, the quantities of gluconolactone, cytosine, and uric acid were decreased indicating higher consumption than control cells. These results indicate that fibroblasts alter gene expression and mitochondria to compensate for hypoxic stress and maintain viability. Furthermore, the metabolic changes observed, making them more similar to preimplantation embryos, could be facilitating nuclear reprogramming making these cells more amendable to future use in somatic cell nuclear transfer.

## Introduction

Otto Warburg first characterized the warburg effect in cancer cells as a metabolic phenomenon, where even in the presence of adequate oxygen, there is high glycolytic activity (aerobic glycolysis) and lactate production (Warburg, 1956). The Warburg effect has been described in proliferative cell types, such as cancers, stem cells, yeast, and early embryos (Conant and Wolfe, 2007; Krisher and Prather, 2012; Redel et al., 2012; Thomson et al., 2005; Vander Heiden et al., 2009; Varum et al., 2011). Warburg had speculated that cancer may be the resultant of mitochondrial damage in the metabolic switch from mitochondrial respiration to increased fermentation of sugars; however, it is now evidenced to be a consequence of mutations in tumor suppressors, oncogenes, and mitochondrial DNA (Koppenol et al., 2011; Warburg, 1956).

The inquisition as to whether hypoxia would inhibit mitochondrial oxidative phosphorylation in fibroblasts, thereby inducing higher glycolytic flux similar to that observed in Warburg effect-like metabolism remained unanswered. We hypothesized that subsequent gene expression and media metabolite production would reflect increased glycolytic flux after culture in restricted oxygen. In this study, we investigated effects of hypoxia on fibroblast viability and size as well as mitochondrial measures, such as quantity, size, and membrane potential. Conditioned media metabolites and gene expression were analyzed to determine if metabolic pathways consistent with the Warburg effect were induced in primary fetal fibroblast cells cultured in hypoxic conditions.

## Materials and Methods

All materials and supplies were purchased from Sigma-Aldrich (St. Louis, MO) unless otherwise specified.

### Compliance with ethical standards

This article does not contain any studies with human participants performed by any of the authors. All procedures performed were in accordance with the ethical standards of the University of Missouri Institutional Animal Care and Use Committee at the University of Missouri in Columbia, MO.

### Fetal-derived fibroblast cell culture

For all experiments within this study the fibroblasts used were isolated from the dorsal proportion of a single male day 35 porcine fetus, largely propagated and passaged once, then were cryopreserved in liquid nitrogen (0.5 mL aliquots; ∼80 cryogenic vials). Detailed protocols used in this project to isolate porcine fetal fibroblasts have been reported elsewhere (Mordhorst et al., 2018). In these experiments a male fibroblast line arising from a cross between a miniature boar (available from the National Swine Research and Resource Center; cell line NSRRC: 0027) and a domestic gilt (Landrace and Yorkshire gilt, from the University of Missouri Swine Teaching and Research Farm) was established.

A separate vial of fibroblasts was thawed for each replicate of each experiment. Cells were cultured under oxygen treatments for 7 days in DMEM (1 g/L glucose; Sigma, St. Louis, MO supplemented with 15% fetal bovine serum [Corning, Manassas, VA]) in T25 flasks (Corning, Corning, NY). The medium was changed daily with preequilibrated medium that had been placed in incubators roughly an hour before achieving equilibrated gas concentrations and to prevent cell shock. During experiments, incubators were maintained at 38.5°C with a humidified atmosphere of 5% carbon dioxide.

Cells were either treated as control (CON) cultured in 5% oxygen for 7 days or cultured in step-wise decreasing concentrations of oxygen (hypoxia; HYP), where for 2 days they were maintained at 5% oxygen, on the third day cultured at 2.5% oxygen, and from the fourth to the seventh days cultured at 1.25% oxygen. Decreased oxygen concentrations in incubators were achieved by increased nitrogen concentration. The concentration of oxygen was regularly monitored by a handheld device with an error of ±0.5% oxygen. The oxygen probe was left in the incubator and the line was accessed without opening the incubator door.

All cells were passaged after 5 days of growth and diluted to achieve even numbers (5 × 10^5^ cells per T25 flask or six-well plate) across treatments by using an automated cell counter. For passaging, dissociation reagents were kept cold. At passaging, flasks were briefly rinsed with PBS +0.01 M EDTA, and fibroblast cells were dissociated from flasks by brief incubation (37°C) with 1 × TrypLE Express (Gibco, Denmark). Cells were replated in media, which were equilibrated in the respective treatment incubators for ∼1 hour. We took great effort to mitigate exposure to oxygen whenever possible when we were transporting cells to be analyzed by: equilibrating media, using plug-sealed cap flasks, covering six-well plates in parafilm, and transporting as quickly as possible in styrofoam coolers.

### Mitotracker green flow cytometry acquisition

Fibroblasts were dissociated with Accutase and incubated at 37°C with 100 μm Mitotracker green for 30 minutes. For flow cytometry data collection, gating protocols were applied for analysis of a population free of debris and doublet cells. Three biological replicates were collected for analysis and three technical samples (50,000 or more single cells) for each treatment were analyzed through flow cytometer to provide a mean for each replicate. A flask of cells was considered as one experimental unit. Mean fluorescence intensity in the FL1 (530/40 dichroic filter) channel was measured in MitoTracker Green-stained fibroblast cells by using a Beckman Coulter CyAN ADP Analyzer cytometer (Beckman Coulter, Inc., Fullerton, CA).

A plot of arbitrary event “count” × FL1 intensity was used to determine mean intensity for each of the treatment replicates acquired. Unstained controls were used to determine positive fluorescence intensity in the FL1 intensity plot. Data were confirmed to be normally distributed as measured through UNIVARIATE procedure in SAS 9.3 (SAS, Cary, NC), which included the following normality tests: Shapiro–Wilk, Kolmogorov–Smirnov, Anderson–Darling, and Cramér–von Mises. Based on these tests, log transformations were made to achieve normality before statistical analysis. Data were analyzed by using the MIXED procedure of SAS 9.3 for main effect of treatment. Differences with a *p*-value of <0.05 were considered significant. Least square mean values are reported with standard errors.

### High-pressure freezing and processing for electron microscopy

For ultrastructural analyses, fibroblasts of both oxygen treatments were grown for 5 days. Afterward, fibroblasts were then dissociated and plated in six-well plates on gold-coated sapphire discs (3 mm in diameter; Wohlwend GmbH, Switzerland) and treated for two additional days. On day 7 of treatments, the cells were cryoimmobilized by high-pressure freezing using the Wohlwend HPF Compact 02 and freeze substituted in acetone containing 0.1% (w/v) uranyl acetate, 0.5% (w/v) osmium tetroxide, and 0.5% (w/v) imidazole.

Freeze substitution was performed in a Leica AFS by raising the temperature from −90°C to 0°C over a period of 18 hours. Samples were infiltrated with Epon and polymerized at 60°C. Seventy-five nanometer-thin sections were prepared, mounted on formvar/carbon-coated copper grids, and subsequently imaged with a JEOL 1400 transmission electron microscope equipped with a CCD camera at an acceleration voltage of 80 kV. Cell features and organelles were outlined and measured using ImageJ (Schneider et al., 2012).

For each of the three biological replicates, at least three cells were measured as technical replicates; the average was six measured cells for each treatment of each replicate. We considered the well in which multiple disks were cultured to be the experimental unit. Data from these measures were analyzed for main effect of oxygen (low oxygen or control) with incorporation of random effects for replicate. Model assumptions, such as normality, constant variances, and linearity, are assessed by SAS output as mentioned above. Observations that are severely deviated from model assumptions were transformed to square root or logarithm before modeling. Model fitting and testing are performed by the MIXED procedure in SAS (version 9.4).

### Conditioned media metabolite gas chromatography and mass spectroscopy analysis

Conditioned media samples were collected from three replicates of HYP (1.25%) and CON (5%) oxygen culture flasks on days 3, 5, and 7 of culture and were centrifuged at 600 *g* for 6 minutes to remove any cellular debris, then stored at −20°C. Conditioned medium was thawed, vortexed, and 1 mL was used for metabolite analysis. Chloroform (1 mL) and HPLC-grade water containing internal standard 25 μg/mL ribitol (1 mL) were added to media samples. The samples were then vortexed and centrifuged at 2900 *g* for 30 minutes at 4°C to separate the layers. The upper aqueous layer (1 mL) was collected and transferred to individual 2.0-mL autosampler vials and dried under nitrogen at 45°C.

Dried polar compounds were methoximated in pyridine with 120 μL of 15.0 μg/mL methoxyamine HCl, briefly sonicated, and incubated at 50°C until the residue was resuspended. Metabolites were then derivatized with 120 μL of MSTFA +1% TMCS for 1 hour at 50°C. The samples were subsequently transferred to a 300 μL glass insert and analyzed using an Agilent 6890 gas chromatographer coupled to a 5973 MSD scanning from m/z 50 to 650. Samples were injected at a 15:1 split ratio, and the inlet and transfer line were held at 280°C. Separation was achieved on a 630 m DB-5MS column (0.25 mm ID, 0.25 μm film thickness; J&W Scientific) with a temperature gradient of 5°C/min from 80°C to 315°C and held at 315°C for 12 minutes, and a constant helium flow of 1.0 mL/min.

The raw data were processed by using AMDIS software (Automated Mass spectral Deconvolution and Identification System, http://chemdata.nist.gov/mass-spectra/amdis/). Derivatized metabolites were identified by matching retention time and mass spectra to those in a custom library of authentic compounds. Abundances of the metabolites were extracted with MET-IDEA (Broeckling et al., 2006; Lei et al., 2012), and then normalized to the abundance of the internal standard (ribitol) for statistical analyses. Conditioned media metabolite quantities were analyzed by using the program SAS (version 9.3; SAS). Flask from which media came from was considered the experimental unit.

The model for each of the metabolites included treatment effect (HYP or CON) and day effect (3, 5, or 7) as fixed effects, and the replicate as a random effect. The interaction between oxygen treatment and day was included when significant. The heterogeneous autoregressive (1) or heterogeneous compound symmetry covariance structures were used to model the correlations among the repeated measures at different days. To meet the normality assumption in the linear regression models, the metabolites were either modeled at original scale or transformed to log scale or square root scale.

The studentized residual plot and normal quantile plot were used for checking model fitting. For the pairwise comparisons, the Tukey–Kramer method for multiple test adjustment was used. Differences with a *p*-value of <0.05 were considered significant. Differences with *p*-values ≥0.05 and ≤0.10 were considered as tendencies. Least square mean values are reported with standard errors unless otherwise stated.

### Extraction of RNA and sequencing

A confluent T25 flask of fibroblasts was collected for RNA after 7 days of respective culture treatments for four biological replicates. The cells were dissociated from culture flasks by brief incubation (37°C) with 1 × TrypLE Express (Gibco, Denmark) by the same method used for cytometry. Cells were pelleted (5 minutes at 500 × G), rinsed with 1 × PBS, and again pelleted. The pelleted cells were plunged into liquid nitrogen and stored at −80°C. Extraction of RNA was performed as per specifications by using the Qiagen RNeasy Mini Kits (Qiagen, Germantown, MD).

Total RNA quality was determined at the University of Missouri DNA core facility by using the Advanced Analytical Fragment Analyzer, and RNA quality scores were assigned based on (1) the presence of discrete 18S and 28S rRNA bands, (2) the mass ratio between the 28S and 18S rRNA, (3) the absence of fragments in the pre-18S and 28S regions, and (4) absence of contaminating high-molecular-weight fragments. All RNA utilized in this study had scores of 10 (the highest score possible). RNA-seq libraries were prepared at the University of Missouri Core facility by using standard Illumina protocol and sequenced on an Illumina HiSeq 2000 platform as single-end reads with read depth of 50 million reads/sample.

The raw sequences (FASTQ) were subjected to quality check by FastQC (www.bioinformatics.babraham.ac.uk/projects/fastqc/). The program fqtrim (https://ccb.jhu.edu/software/fqtrim/) was used to remove adapters, perform quality trimming (phred score >30) by a sliding window scan (6 nucleotides), and select read length of 30 nucleotides or longer after trimming.

Reads obtained from the quality control step were mapped to the Sus Scrofa (v10.2) reference genome by using Hisat2 aligner, which is a fast and sensitive alignment program of next-generation sequencing data (Kim et al., 2015). The program FeatureCounts (Liao et al., 2014) was used to quantify read counts by using the sequences alignment files of each sample. The differentially expressed (DE) genes between sample groups, representing the culture treatment, were determined by edgeR-robust (Zhou et al., 2014). The false discovery rate <0.05 was used as threshold for the statistically significant differential expression of genes. The flask from which cells came from was considered to be the experimental unit in this analysis.

## Results

### Impact of treatment on fibroblast viability and mitochondrial staining intensity

Positive Annexin-V-FITC and Propidium Iodide staining was used to detect apoptotic and dead cells, respectively. The percentages of viable and dead cells were not significantly different between HYP and CON cells (*p* ≥ 0.23; Table 1). The small percentage of cells undergoing Annexin-V membrane exposure (indicative of early apoptosis) tended to be larger in hypoxic cells than control (1.70% vs. 1.11% ± 0.24%; *p* ≥ 0.06; Table 1). The percentage of cells which was considered to be in late apoptosis (stained singly for Propidium Iodide) was greater (*p* = 0.02) in CON cultured cells than HYP (0.55% vs. 0.29% ± 0.16%; Table 1) although it was less than 1% of fibroblasts analyzed.

**Table 1.** Viability of Fibroblasts After 7-Day Culture Under Hypoxia (Restricted Oxygen Gradient Culture; 5% −1.25%) or Control (5%) Oxygen Concentrations

	*Oxygen treatment*			
	*Control*	*Hypoxia*	*SE*	p
Healthy^a^	95.18	96.25	1.24	0.34
Early apoptotic^b^	1.11	1.70	0.24	0.06
Late apoptotic^c^	0.55	0.29	0.16	0.02
Necrotic^d^	3.20	1.76	1.30	0.23

^a^ Percentage of fibroblast population, which stained negative for Annexin-V-FITC and Propidium Iodide.

^b^ Percentage of fibroblast population, which stained positive for Annexin-V-FITC and negative for Propidium Iodide.

^c^ Percentage of fibroblast population, which stained negative for Annexin-V-FITC and positive for Propidium Iodide.

^d^ Percentage of fibroblast population, which stained positive for Propidium Iodide and positive for Annexin-V-FITC.

Intensity of MitoTracker fluorescence intensity was greater (*p* < 0.0001) in HYP compared with CON (1097.3 vs. 668.8 ± 22.9 AU).

### Gene expression

**FIG. 1.**
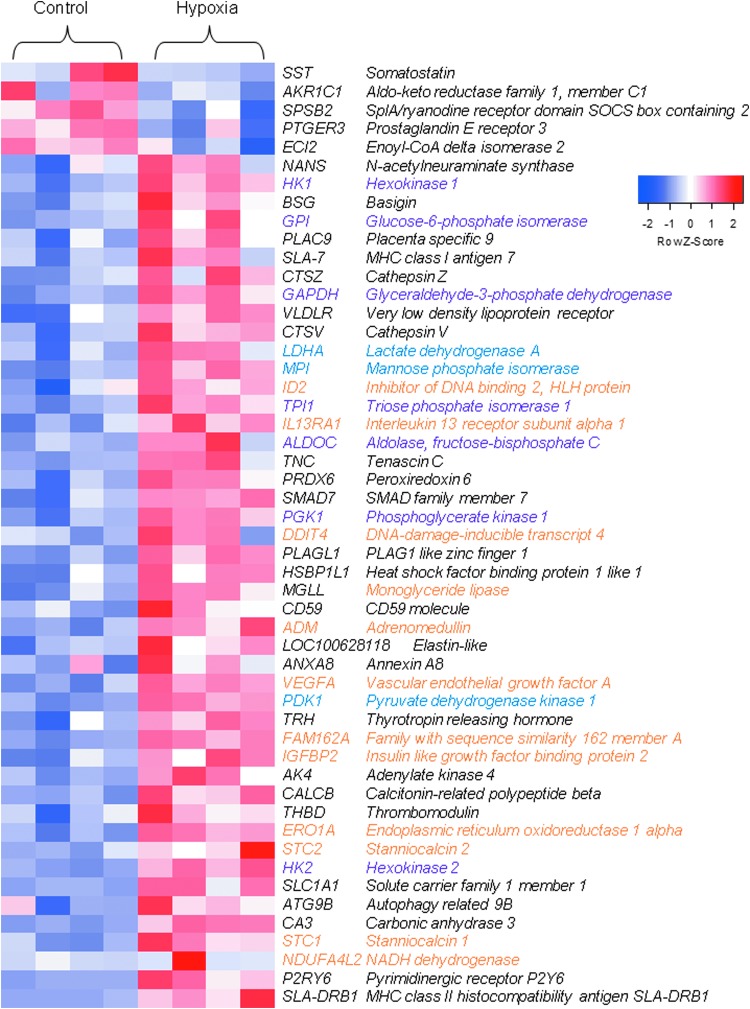
A heat map of differentially expressed (DE) genes between fibroblasts cultured for 1 week in hypoxia (HYP; 2 days in 5%, 1 day in 2.5% and 4 days in 1.2% oxygen) or as controls (CON; 5% oxygen for 7 days). Gene names are color coded: *purple*—upregulated glycolytic/gluconeogenic pathway enzymes; *blue*—upregulated enzymes, which contribute to glycolytic and gluconeogenic metabolism; *orange*—genes previously reported to be induced by hypoxia. The z-score scale of gene differential expression is shown. Four biological replicates with >5 × 10^5^ fibroblasts were used for RNA sequencing from each treatment. Color images available online at www.liebertpub.com/cell

**FIG. 2.**
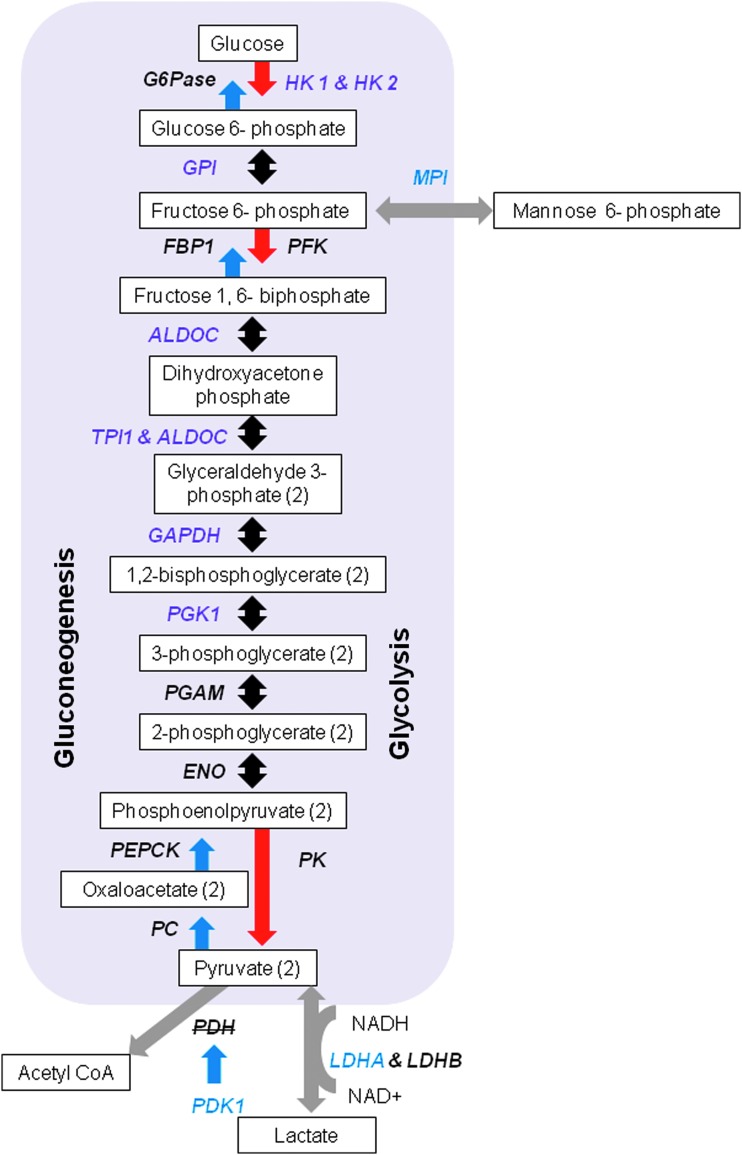
Functional annotation of genes activated by hypoxia involved in glycolytic/gluconeogenic metabolism. Genes for enzymes directly involved in glycolysis and gluconeogenesis, which increased in expression with hypoxic culture are listed in *purple*. Genes for enzymes, which contribute to glycolytic and gluconeogenic metabolism are listed in *blue*. Genes in *black font* were not DE. Genes in strikethrough font indicate inhibition. Outlined *white rectangles* are relevant metabolites. *Black arrows* indicate reversible reactions between glycolysis and gluconeogenesis. *Blue arrows* indicate reactions in gluconeogenesis, and *red arrows* indicate reactions in glycolysis. *Gray arrows* depict reactions outside of the glycolytic and gluconeogenic pathways. Sequenced RNA was collected from fibroblasts cultured for 7 days in hypoxia (HYP; 2 days in 5%, 1 day in 2.5% and 4 days in 1.25% oxygen) or as controls (CON; 5% oxygen for 7 days). Four biological replicates with >5 × 10^5^ fibroblasts were used from each treatment. Color images available online at www.liebertpub.com/cell

Sequencing of mRNA revealed 51 DE genes between HYP and CON treatments (Fig. 1). Of the 51 genes, 5 genes were downregulated and DE in HYP versus CON. In order of fold change in expression, those genes are somatostatin; aldo-keto reductase family 1, member C1; splA/ryanodine receptor domain and SOCS box-containing 2; prostaglandin E receptor 3; and enoyl-CoA delta isomerase 2. The functional annotation of DE genes further showed that seven genes within the glycolysis/gluconeogenesis pathway were activated in response to hypoxia (Figs. 1 and 2, purple font). Additionally, three other genes, which contribute to glycolytic and gluconeogenic metabolism were also activated (Figs. 1 and 2, blue font). Several other DE genes identified from the current study were also previously reported to be induced by hypoxia in other cell types (Fig. 1, orange font); these are elaborated on in the Discussion.

### Impact of treatment on fibroblast ultrastructure

Electron microscopy was used to count and measure cells and mitochondria on thin sections of monolayer cells. The thin sections were obtained within an area of 1–1.5 μm above the substrate, parallel to the surface of the cell monolayer. Area and perimeter of fibroblasts as well as their nuclei were not impacted by oxygen restriction during culture (*p* ≥ 0.20; Table 2). Percentage of the cell that was occupied by the nucleus and the percentage of mitochondrial area in the cytoplasm were also not altered when cells were cultured in hypoxia (*p* ≥ 0.20; Tables 2 and 3).

**Table 2.** Ultrastructural Cell Features of Fibroblasts Cultured for 7 Days Under Hypoxia (Restricted Oxygen Gradient Culture; 5% −1.25%) or Control (5%) Oxygen Concentrations

	*Treatment*			
*Ultrastructural measures*	*Control*	*Hypoxia*	*SE*	p
Cell perimeter size (μm)	102.3	110.56	24.66	0.64
Cell area^a^ (μm^2^)	375.6	475.51	163.87	0.34
Nucleus perimeter size (μm)	28.0	25.53	6.48	0.20
Nucleus area^b^ (μm^2^)	54.2	53.60	19.51	0.93
Nucleus proportion of cell^c^ (%)	16.9	15.38	2.33	0.54

^a^ Area within cell perimeter.

^b^ Area within perimeter of nucleus.

^c^ Percent of cell area that was nucleus.

**Table 3.** Mitochondrial Parameters of Fibroblasts Cultured for 7 Days Under Hypoxia (Restricted Oxygen Gradient Culture; 5% −1.25%) or Control (5%) Oxygen Concentrations

	*Treatment*			
*Ultrastructural measures*	*Control*	*Hypoxia*	*SE*	p
Total mitochondrial area^a^ (μm^2^)	5.2	4.7	1.71	0.44
Mitochondrial proportion of cell^b^ (%)	3.20	10.31	6.20	0.84
Mitochondrial number^c^ (#)	10.69	14.01	0.34	0.0009
Average mitochondrial perimeter size (μm)	2.26	1.89	0.17	<0.0001
Average mitochondrial area^d^ (μm^2^)	0.22	0.16	0.01	<0.0001

^a^ Sum area of all mitochondria within a cell section measured.

^b^ Percent of total mitochondrial volume within cell area–nucleus area in section.

^c^ Average number of mitochondria within a cell section.

^d^ Average area within average mitochondrial perimeter in a section.

The number, perimeter size, and area of mitochondria were significantly impacted by HYP culture treatment (*p* ≤ 0.0009; Table 3). The number of mitochondria per cell was increased with HYP treatment compared with CON (14.01 vs. 10.69 ± 0.34 mitochondria; Table 3). However, CON cells had larger mitochondrial area and perimeters than HYP (area = 0.22 vs. 0.16 ± 0.01 μm^2^; perimeter = 2.16 vs. 2.39 ± 0.35 μm; Table 3).

### Conditioned cell culture media metabolites

Fifty metabolites were detected in conditioned culture media through tandem gas chromatography–mass spectroscopy. There was a tendency (*p* = 0.06) for the quantity of gluconolactone to be decreased in the media from HYP cell culture compared with control (121.6 vs. 200.6 ± 73.2 AU). Both of these values are increased from the quantity of gluconolactone in unconditioned media (40.2; standard deviation = 20.1 AU). The interaction of day (media collected on days 3, 5, and 7) and oxygen culture treatment (HYP vs. CON) was significant (*p* < 0.05) for uric acid and cytosine.

The level of cytosine remained similar throughout the week in media from HYP cultured cells (day 3 = 2.3 ± 0.5 AU; day 7 = 2.6 ± 1.5 AU), but had increased in CON (day 3 = 2.1 ± 0.5 AU; day 7 = 7.2 ± 1.5 AU). The quantity of cytosine in media from HYP fibroblasts throughout the week was similar to the quantity in unconditioned media (1.9; standard deviation = 1.8 AU). Uric acid quantity in unconditioned media was 41.5 AU (standard deviation = 4.1 AU). On day 3 both treatments had similar concentrations (HYP = 44.2; CON = 46.8 ± 2.1 AU). By day 7, CON had greater uptake compared with HYP, and thereby a lower concentration in conditioned media (8.3 vs. 34.8 ± 3.6 AU). Of the other metabolites detected, the quantities within HYP versus CON-conditioned media were not different (*p* > 0.10).

## Discussion

This study sought to investigate if hypoxia could elicit characteristics of the Warburg effect-like phenotype observed in other cell types, such as preimplantation embryos, in fetal-derived fibroblast cells. First, we wanted to determine if classically hypoxia-inducible cellular changes were occurring in treated fibroblasts. Second, if there was evidence of Warburg effect-like characteristics, such as increased use of glycolysis over tricarboxylic acid cycle (TCA) and the immature mitochondrial appearance observed in early embryos. Overall, we thought this information would provide us with a good indication as to whether hypoxia alone would be enough to drive changes in fibroblast metabolism to a more embryo-like state, which may help facilitate nuclear reprogramming during somatic cell nuclear transfer.

### Canonical hypoxic signaling and cellular morphology present in fibroblasts

A review of cancer by Wouters and Koritzinsky (2008) highlights two hypoxia signaling pathways: regulation of the mammalian target of rapamycin (mTOR) and the unfolded protein response (UPR). In this study we measured changes in mRNA expression by RNA sequencing of HYP and CON treated fibroblasts after 1 week of culture. Increased expression of DNA damage-inducible transcript 4 protein (*DDIT4*; also known as *REDD1*) and endoplasmic reticulum oxidoreductase 1 alpha (*ERO1A*) in HYP fibroblasts evidence that these pathways may have been stimulated. Both genes were previously evidenced to be expressed under hypoxic stress (Gess et al., 2003; Jin et al., 2007; Shoshani et al., 2002). The mTOR pathway is inhibited by *DDIT4*, thereby regulating cell growth and survival; which may in part explain why HYP cells had no differences in size or viability from CON.

**FIG. 3.**
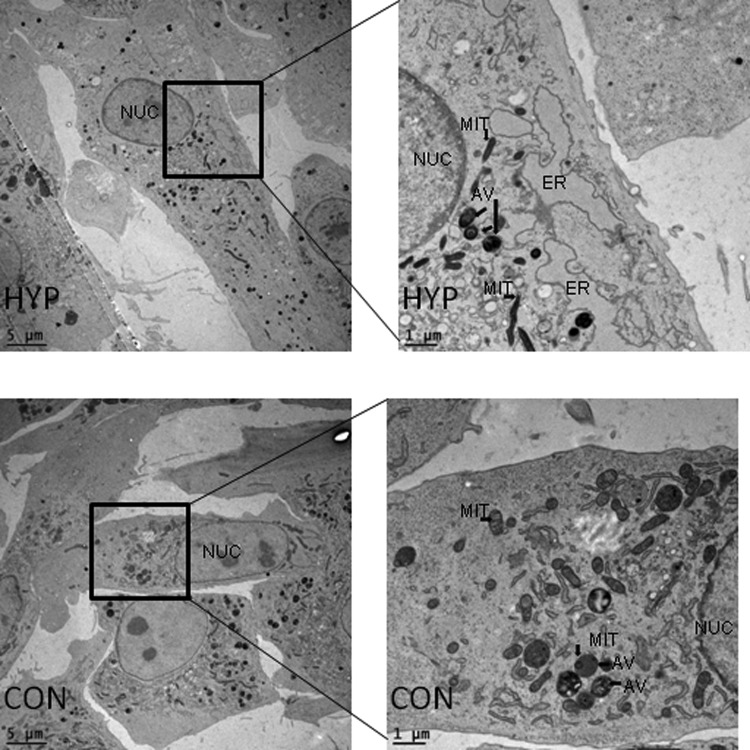
Electron micrographs of fibroblasts cultured for 7 days in hypoxia (HYP; 2 days in 5%, 1 day in 2.5% and 4 days in 1.2% oxygen) or as controls (CON; 5% oxygen for 7 days). For analysis, three biological replicates with at least three cells were measured as technical replicates; the average was six measured cells for each treatment of each replicate. MIT, mitochondria; NUC, nucleus; ER, endoplasmic reticulum; AV, autophagic vesicle.

In addition to expression of *ERO1A*, a gene essential for the maintenance of endoplasmic reticulum redox homeostasis and protein folding activity during stress, we observed a “stressed” appearance in endoplasmic reticulum from HYP fibroblasts (Fig. 3).

Expression of calcium homeostatic regulators stanniocalcin 1 and 2 (*STC1* and *STC2*; Fig. 1; orange font) were elevated in HYP cells compared with CON. Evidenced to be induced by hypoxic stress (Westberg et al., 2007; Yeung et al., 2005), *STC1* plays a role in antioxidant and anti-inflammatory cellular defense (Kim et al., 2013; Sheikh-Hamad, 2010; Tang et al., 2014). Also activated during hypoxia, *STC2* is characterized to be specifically targeted by the UPR (Ito et al., 2004; Zeiger et al., 2011). Hypoxic tension has been linked to ER stress and autophagy (Bi et al., 2005; Pereira et al., 2014; Ye and Koumenis, 2009). In HYP cells, expression of autophagy-related 9B (*ATG9B*), was increased compared with CON; this protein organizes early autophagosome structure by facilitating cytoplasm- to-vacuole transport and vesicle formation.

Autophagic vesicles were observed in both CON and HYP cells; however, not directly quantified due to the challenges of correctly deciphering the autophagic vesicles from other vesicle types present without proper immunomarkers (Fig. 3). Collectively, these results evidence that comparable to other cell types experiencing hypoxia, porcine fetal fibroblasts activate similar canonical signaling pathways and display typical hypoxic cellular morphology.

### Hypoxia-induced metabolic changes in fibroblasts

Under hypoxic stress, cells can induce autophagy and mitophagy to promote survival, which is achieved through activation of hypoxia-inducible factor (*HIF-1*) (Hu et al., 2012; Mazure and Pouysségur, 2010). An important well-studied modulator of oxygen homeostasis and survival, *HIF* activates the transcription of genes for metabolic adaptation to hypoxia, including glycolytic enzymes, glucose transporters, and angiogenic genes (Bergeron et al., 1999; Greijer et al., 2005; Jiang et al., 1997; Semenza, 2000).

We did not detect a statistical difference in *HIF* expression, however, we did see increased expression of several established *HIF*-activated downstream genes (Fig. 2, orange font), including vascular endothelial growth factor A (*VEGFA*), interleukin 13 receptor subunit alpha 1 (*IL13RA1*), family with sequence similarity 162 member A (*FAM162A*), insulin-like growth factor-binding protein 2 (*IGFBP2*), inhibitor of DNA-binding 2, HLH protein (*ID2*), stanniocalcin (*STC1*), stanniocalcin (*STC2*), and adrenomedullin (*ADM*), as well as seven glycolytic enzymes (Figs. 1 and 2, purple font) in HYP cultured cells (Bergeron et al., 1999; Choi et al., 2008; Copple et al., 2011; Feldser et al., 1999; Jean et al., 2006; Jiang et al., 1997; Leonard et al., 2003; Löfstedt et al., 2004).

A study comparing impacts of hypoxia on transcription of synovial healthy and rheumatoid arthritic fibroblasts found that in both cell lines hypoxia upregulated expression of *VEGFA*, *NDUFA4L2* (NADH dehydrogenase 1 alpha subunit, 4-like 2), *ALDOC* (Aldolase, fructose-bisphosphate C), *PGK1* (Phosphoglycerate kinase 1), and *STC1* similar to fibroblasts in our study (Del Rey et al., 2010).

A limitation we have is that some of the glycolytic genes expressed are reversible enzymes, which are used in both glycolysis and gluconeogenesis. However, increased expression of hexokinase 1 and 2 in HYP fibroblasts might indicate glycolytic activity to be the predominating pathway. In addition, expression of *Mannose phosphate isomerase* was increased in HYP cells (Figs. 1 and 2, blue font); this enzyme catalyzes the conversion of mannose-6-phosphate to fructose-6-phosphate, a metabolite in the glycolytic pathway.

Moreover in HYP fibroblasts, expression of lactose dehydrogenase A (*LDHA*) was increased compared with the control (Figs. 1 and 2; blue font). Gluconeogenesis is useful in recycling lactate and pyruvate when cells are in a hypoxic state. Since we did not see differences in the metabolite production of alanine, pyruvate, or lactate between HYP and CON cells, this may therefore indicate activation of gluconeogenesis to support increased gluconeogenesis.

Papandreou et al. (2006) evidenced hypoxic adaptation, whereby, in addition to increased expression of glycolytic enzymes, mitochondrial oxygen consumption was decreased through downstream *HIF* activation of pyruvate dehydrogenase kinase 1 (*PDK1*). This kinase functions to inactivate pyruvate dehydrogenase, the enzyme which facilitates entry of pyruvate into the TCA, thereby decreasing mitochondrial oxidative phosphorylation. Expression of PDK1 was increased in HYP cells compared with CON (Fig. 1; blue font). Conditioned media collected throughout the week were processed for metabolites by using tandem gas chromatography–mass spectroscopy. While both HYP and CON cells produced gluconolactone, an intermediate between glycolysis and the pentose phosphate shunt, CON media had higher quantities than HYP.

The amount of cytosine in HYP cells was similar to the initial quantities found in unconditioned media, however, in CON cells cytosine had increased. These results may indicate that while both HYP and CON cells were producing these metabolites, HYP cells may have had increased need and usage of metabolites perhaps for the synthesis of nucleotides through the pentose phosphate pathway. In day 7 conditioned media, the concentration of uric acid was higher in HYP cells than CON; however, they were both lower than the quantity present in unconditioned media.

Uric acid is a recognized antioxidant (Ames et al., 1981). *In vivo*, uric acid was shown to protect rat neurons and restore mitochondrial function after insult (Yu et al., 1998). Uric acid concentration may have been lower in CON cells because they had oxygen available to oxidize it, whereas in HYP cell, the inability to oxidize uric acid may have caused a buildup. One limitation to measuring metabolites in media is that the intracellular concentration and production is unknown.

### Hypoxic culture increased mitochondrial quantities in fibroblasts

Electron microscopy was employed to establish whether hypoxia impacted the quantity or relative size of mitochondria in cultured monolayers. The relative total mitochondrial area among HYP and CON fibroblasts was not different; however, HYP fibroblasts on average had more mitochondria (a 31% increase) that were smaller in area (37.5%) and perimeter (19.6%; Table 3) within the section taken from the monolayer.

However, the morphology of mitochondrial cristae appeared similar to CON in HYP fibroblasts (Fig. 3). The surface area and perimeters of our fibroblasts (Table 2) are similar to the findings of others using human fibroblast cell lines (Barbucci et al., 2005; Brugmans et al., 1983). There were no statistical differences in cell size or viability between CON and HYP cultured cells. Comparably, in human cardiac fibroblasts, 2% oxygen did not alter cell viability; however, it did impair protein synthesis (Agocha et al., 1997).

## Conclusions

The current study is novel in its approach to program metabolism of donor fibroblasts to a Warburg effect-like state similar to that of preimplantation embryos in an effort to improve somatic cell nuclear transfer. To date, we do not know of any other groups which have tried similar strategies outside of our laboratory (Mordhorst et al., 2018a, 2018b).

Over the years, there have been several attempts to enhance reprogramming of donor cells, including, but not limited to, investigations of serum starvation and cell cycle regulation, type of cell, age of animal from which cells were extracted, age of cells themselves (or passage number), epigenetic reprogramming, degree of pluripotency, and antioxidant treatment of cells (Bonk et al., 2007; Campbell et al., 1996; Chen et al., 2015; Dominko et al., 1999; Heyman et al., 2002; Iager et al., 2008; Kato et al., 2000; Mitalipov et al., 2002; Oback and Wells, 2007; Powell et al., 2004; Tani et al., 2001; Wakayama and Yanagimachi, 2001; Wells et al., 2003; Whitworth et al., 2011; Wilmut et al., 2002; Yang et al., 2012).

To date, while somatic cell nuclear transfer efficiency has improved in other species, none of these treatments has served as the breakthrough needed to dramatically improve the efficiency of somatic cell nuclear transfer in pigs. In swine, the cloning efficiency is reported to be 1–4%, where the majority of transfers fall between 1% and 2% (Kurome et al., 2013; Whitworth and Prather, 2010).

The results from this study indicate that fibroblasts alter gene expression and mitochondrial quantities to compensate for hypoxic environment and maintain viability. The increased transcript abundance of glycolytic genes in addition to expression of PDK-1 implies that glycolysis was favored over mitochondrial oxidative phosphorylation. Therefore, changes in usage of the two central metabolic pathways observed in Warburg effect-like metabolism appear to also be inducible by hypoxia.

Notably, the likely metabolic change in this study is by definition anaerobic glycolysis as the Warburg effect occurs in the presence of oxygen, whereas in this study oxygen restriction, or providing an anaerobic environment, induced changes in metabolism. Regardless, questions posed in these experiments warrant further research. Determining the true metabolic flux occurring between glycolysis, gluconeogenesis, and the pentose phosphate pathways in oxygen-deprived fibroblasts may be useful in understanding how similar the metabolism induced is to that of preimplantation embryos.

Further investigations into whether hypoxia can induce increased metabolic changes in other cell types used in somatic cell nuclear transfer may be beneficial as a number of studies indicate that there is a correlation between increased glycolytic metabolism and pluripotency (Folmes Clifford et al., 2011; Moussaieff et al., 2015; Shyh-Chang and Daley, 2015). Determining whether programming of a Warburg effect-like metabolism similar to early embryos does lead to reprogramming of the nucleus through driving essential changes in gene expression in other cell types may be critical to improving the efficiency of somatic cell nuclear transfer.
